# Identification of plant leaf diseases by deep learning based on channel attention and channel pruning

**DOI:** 10.3389/fpls.2022.1023515

**Published:** 2022-11-10

**Authors:** Riyao Chen, Haixia Qi, Yu Liang, Mingchao Yang

**Affiliations:** ^1^ College of Engineering, South China Agricultural University, Guangzhou, China; ^2^ National Center for International Collaboration Research on Precision Agricultural Aviation Pesticides Spraying Technology, Guangzhou, Guangdong, China; ^3^ Guangdong Laboratory for Lingnan Modern Agriculture, Guangzhou, Guangdong, China; ^4^ College of Horticulture, South China Agricultural University, Guangzhou, China

**Keywords:** CACPNET, deep learning, plant leaf disease, convolutional neural network, channel attention, channel pruning

## Abstract

Plant diseases cause significant economic losses and food security in agriculture each year, with the critical path to reducing losses being accurate identification and timely diagnosis of plant diseases. Currently, deep neural networks have been extensively applied in plant disease identification, but such approaches still suffer from low identification accuracy and numerous parameters. Hence, this paper proposes a model combining channel attention and channel pruning called CACPNET, suitable for disease identification of common species. The channel attention mechanism adopts a local cross-channel strategy without dimensionality reduction, which is inserted into a ResNet-18-based model that combines global average pooling with global max pooling to effectively improve the features’ extracting ability of plant leaf diseases. Based on the model’s optimum feature extraction condition, unimportant channels are removed to reduce the model’s parameters and complexity *via* the L1-norm channel weight and local compression ratio. The accuracy of CACPNET on the public dataset PlantVillage reaches 99.7% and achieves 97.7% on the local peanut leaf disease dataset. Compared with the base ResNet-18 model, the floating point operations (FLOPs) decreased by 30.35%, the parameters by 57.97%, the model size by 57.85%, and the GPU RAM requirements by 8.3%. Additionally, CACPNET outperforms current models considering inference time and throughput, reaching 22.8 ms/frame and 75.5 frames/s, respectively. The results outline that CACPNET is appealing for deployment on edge devices to improve the efficiency of precision agriculture in plant disease detection.

## Introduction

Each year about 30 percent of global crop yields are lost due to plant diseases, resulting in direct economic losses exceeding 40 billion dollars (Dong et al., 2021). More than 821 million people have suffered from food insecurity in the past five years (Krishnamurthy et al., 2020). There are many diseases responsible for a series of losses, among which leaf spot is a common disease that often occurs in crops such as rice (Harish et al., 2008), maize (Barupal et al., 2020), and peanuts (Qi et al., 2021). Therefore, accurate identification and timely diagnosis of plant diseases are significant for plant protection (Singh and Misra, 2017). In practice, disease identification depends on professionals imposing high labor costs, lack of real-time monitoring, and unprofessional misidentification, which further increase the difficulty of identifying diseases in agriculture and lead to unstable and sharp declines in yields and food security problems (Ferentinos, 2018). Thus, intelligent and accurate identification of plant diseases without relying on manpower remains challenging for the precision agriculture field (Donatelli et al., 2017).

Recent advances in computer technology afford image classification, object detection, and natural language processing using deep learning (Li et al., 2018; Sharma and Mir, 2020; Otter et al., 2021). Currently, several deep neural network (DNN) models have been developed based on CNN features for image feature extraction, e.g., AlexNet, VGG, ResNet, and DenseNet (Krizhevsky et al., 2012; Simonyan and Zisserman, 2014; He et al., 2016; Huang et al., 2017).

Due to the powerful feature extraction capability of deep learning, researchers have already applied the above models to plant disease identification (Dhaka et al., 2021). For instance, an improved AlexNet model was used on rice diseases achieving a recognition accuracy of 95.4% (Lu et al., 2017). VGG was used on cucumber diseases after improving the fully connected layer (Zhang et al., 2019). Furthermore, ResNet-50 identified the grapevine yellows symptoms (Cruz et al., 2019), GoogLeNet was applied for disease identification in maize, tomato, and eggplant (Li et al., 2020; Pan et al., 2022), while DenseNet was used to classify nutrient deficiencies in rice crop (Sathyavani et al., 2021). The studies above demonstrate that deep neural networks improve plant disease recognition accuracy but still impose an extremely high computational cost because the models have many parameters. Specifically, the VGG-16 model has 138 million parameters and requires 15.484 Giga Floating Point Operations (GFLOPs) to conduct image recognition (Simonyan and Zisserman, 2014). The above models containing a large number of parameters are not efficient to run on plant protection equipment with limited computing power such as unmanned aerial vehicle and robots. Furthermore, due to the complexity of the field environment and the similarity of plant diseases, identification errors may lead to the spread of plant diseases. Accordingly, the recognition accuracy of the above study cannot meet the requirements of precision agriculture. Based on these results, directly applying DNN models to plant disease identification may not be effective. Therefore, enhancing the neural network’s feature extraction capability and compressing the DNN models have become two significant challenges for precision agriculture to apply deep learning in the plant disease identification field.

Recently, to improve the model’s recognition accuracy in large-scale classification tasks, the attention module has achieved remarkable results (Woo et al., 2018; Hu et al., 2019). In agriculture, the CBAM attention module based on DenseNet was utilized for wheat stripe rust recognition, which improved the accuracy rate by 5.47% compared with a native model (Mi et al., 2020). The ResNet-50 with SENet attention module has also been used to identify vegetable diseases with 97.24% accuracy after employing transfer learning (Zhao et al., 2022). The above results reveal that the attention module can effectively improve recognition accuracy but increases the computational time to process a single image. Therefore, the attention module is inefficient and suffers from a computationally intensive and complex structure. To balance the relationship between performance and complexity, an efficient channel attention mechanism module called ECA was proposed (Wang et al., 2020). The ECA module significantly improves the model’s recognition accuracy by adding only a few parameters. Indeed, the crop disease model based on the ECA module was validated on the AI Challenger 2018 dataset, PlantVillage dataset, and self-collected cucumber disease dataset, attaining recognition accuracies of 86.35%, 99.74%, and 98.54%, respectively (Gao et al., 2021). Although the attention module improved the recognition accuracy of crop diseases, it did not reduce the model’s parameter redundancy in the feature extraction process. Nevertheless, models with complex structures and excessive parameters impose significant hardware resource consumption and reduce recognition efficiency. Therefore, developing a model that achieves high accuracy while being sufficiently lightweight is still a challenge in plant disease recognition.

With advances in the Internet of Things and machine vision, mobile platforms such as unmanned aerial vehicle and robots make precision agriculture develop quickly (Tang et al., 2020; Bouguettaya et al., 2022). Due to the conflict between the high computational power requirements of the models and the limited computational power of plant protection equipment, it is a challenging task to deploy plant disease detection models on mobile platforms (Neupane and Baysal-Gurel, 2021). Currently, mobile devices are mostly used as a means of image acquisition, with disease images being transferred to more capable devices for identification (Xenakis et al., 2020). Nevertheless, recent research highlights that image recognition can be achieved using shallow networks as well (Kundu et al., 2021; Wieczorek et al., 2022), with model pruning being an effective model compression method whose core strategy is reducing the DNN’s complexity *via* discarding redundant and uninformative weights (Han et al., 2015). After pruning, the model achieves an apparent acceleration while being lightweight. Adding sparse constraints in the training stage can reduce the model’s number of neurons and thus reduce the parameters and memory occupation (Zhou et al., 2016). However, the recognition accuracy can be significantly reduced due to discarding important parameters (Guo et al., 2020). Hence, it should be noted that a valid channel pruning metric must reduce the impact on model accuracy and consider the channel’s importance in different layers.

In the precision agriculture field, deep learning is widely used in plant disease detection, but it still faces the problems of inefficient accuracy and excessive computational cost. In addition, the recognition rate is also an issue worthy of attention while applying the model to real-time detection of plant diseases in the field. The attention mechanism can effectively improve the identification accuracy of the model, while DNN complexity increases when adding an attention module. The ECA module uses a local cross-channel interaction strategy without dimensionality reduction, which improves accuracy without bringing in a massive quantity of parameters. However, the local feature extraction ability of the ECA module is limited and thus unable to extract the features of plant diseases well. In real time detection of plant diseases, the low recognition rate of models is one of the main factors limiting their detection effectiveness. Interestingly, pruning methods can be applied to model compression to achieve model acceleration. However, model pruning may decrease model accuracy. In addition, there is a lack of highly accurate and lightweight models that can be deployed to terminal inspection equipment in plant protection. Therefore, we propose the CACPNET model, which combines channel attention and pruning to solve the above-mentioned problems. The main contributions are summarized as follows:

1. The ECA module is modified to improve the model’s ability to identify diseases for plant leaf diseases.
2. Without a significant loss of the model’s accuracy, the model is channel pruned based on the channel weight importance and the local compression ratio. This strategy affords a highly accurate and lightweight model.
3. Model validation is performed using the public dataset PlantVillage and our peanut leaf disease dataset. The model’s performance is analyzed based on accuracy, F1 score, FLOPs, parameters cardinality, model size, and GPU RAM.
4. The model’s operation is simulated on the plant protection detection equipment, and the model’s recognition rate is analyzed based on inference time and throughput metrics.
5. This study fills the research gap in real-time detection of leaf diseases, including peanuts, potatoes, apples, and other 15 crops and 43 diseases. Meanwhile CACPNET can be used for training and identification of other plant diseases.

## Materials and methods

### Dataset acquisition

This paper utilizes two datasets for experiments, namely the PlantVillage and the peanut leaf disease dataset we collected.

### PlantVillage dataset

This dataset comprises 54634 leaf images divided into 38 disease classes from 14 species: apple, blueberry, cherry, corn, grape, orange, peach, pepper, potato, raspberry, soybean, squash, strawberry, and tomato. The details on the PlantVillage dataset are presented in **Table 1**. The dataset is randomly divided into a training and test set according to a 4:1 ratio with a uniform image resolution of 224×224 pixels.

**Table 1:** Basic information of the PlantVillage.

Crop	Class	Train set	Test set
Apple	Apple_scab	504	126
	Black_rot	497	124
	Cedar_apple_rust	220	55
	Healthy	1316	329
Blueberry	Healthy	1202	300
Cherry	Healthy	684	170
	Powdery_mildew	842	210
Corn	Cercospora_leaf_spot Gray_leaf_spot	411	102
	Common_rust	954	238
	Healthy	930	232
	Northern_Leaf_Blight	788	197
Grape	Black_rot	944	236
	Esca_(Black_Measles)	1107	276
	Healthy	339	84
	Leaf_blight_(Isariopsis_Leaf_Spot)	861	215
Orange	Haunglongbing_(Citrus_greening)	4406	1101
Peach	Bacterial_spot	1838	459
	Healthy	288	72
Pepper	Bacterial_spot	798	199
	Healthy	1183	295
Potato	Early_blight	800	200
	Healthy	122	30
	Late_blight	800	200
Raspberry	Healthy	297	74
Soybean	Healthy	4072	1018
Squash	Powdery_mildew	1468	367
Strawberry	Healthy	365	91
	Leaf_scorch	888	221
Tomato	Bacterial_spot	1702	425
	Early_blight	800	200
	Healthy	1273	318
	Late_blight	1528	381
	Leaf_mold	762	190
	Septoria_leaf_spot	1417	354
	Spider_mites Two-spotted_spider_mite	1341	664
	Target_Spot	1124	280
	Tomato_mosaic_virus	299	74
	Tomato_Yellow_Leaf_Curl_Virus	4286	1071

### Peanut leaf disease dataset

**Figure 1:**
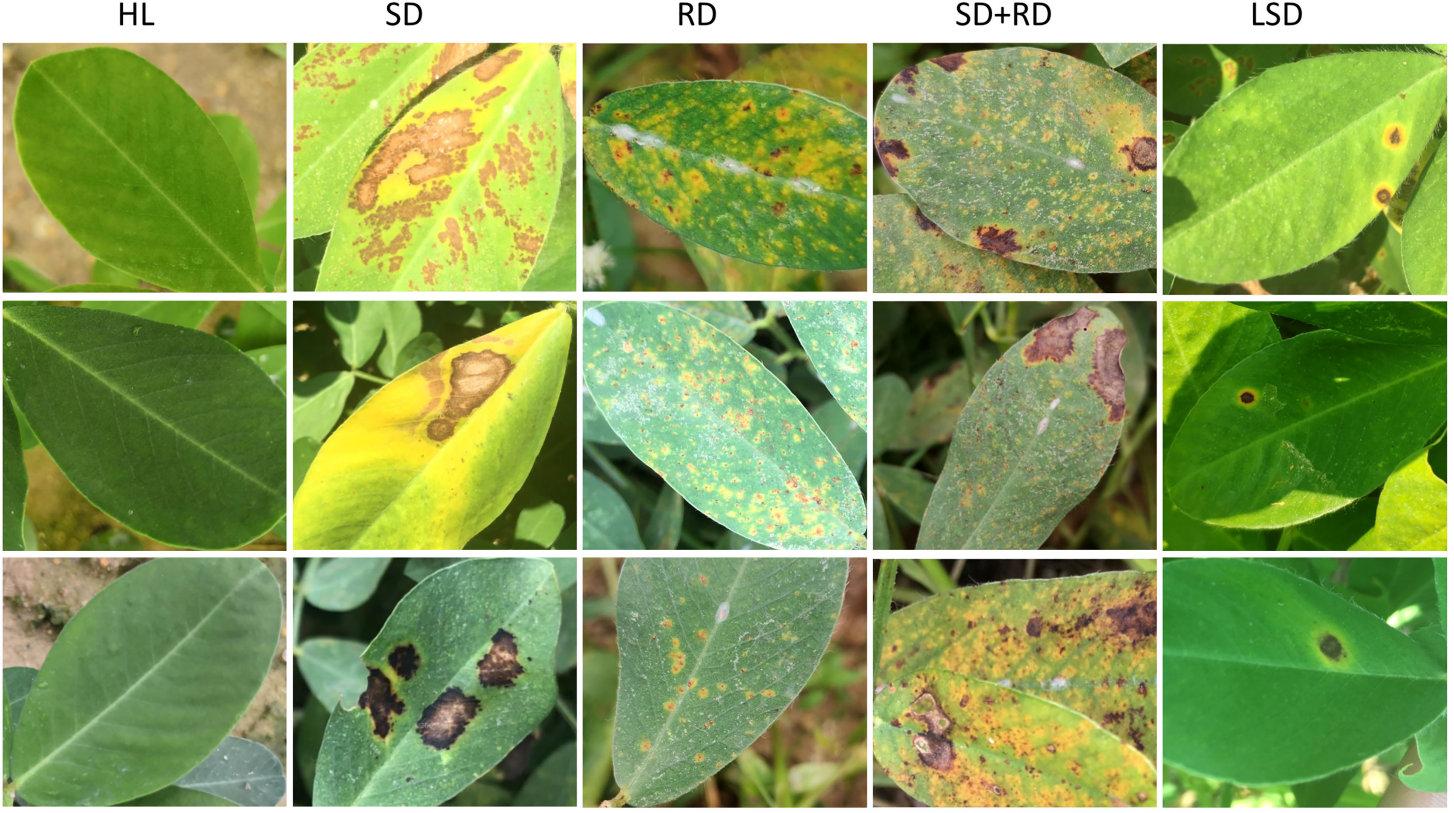
The image of peanut leaf disease. The figures show images from five categories, namely HL (healthy leaves, the first column), SD (scorch disease on a single leaf, the second column), RD (rust disease on a single leaf, the third column), SD+RD (both rust disease and scorch disease on a single leaf, the fourth column), and LSD. (leaf-spot disease on a single leaf, the fifth column).

This is our own collected disease dataset from peanut leaves, collected from the Agronomic experimental base of South China Agricultural University. This dataset has 6033 disease images from five categories, namely healthy leaves (HL), rust disease on a single leaf (RD), leaf‐spot disease on a single leaf (LSD), scorch disease on a single leaf (SD), and both rust disease and scorch disease on a single leaf (SD+RD) (**Figure 1**). The above diseases are common types of diseases in peanuts, which are significant factors causing peanut yield decline.

The images are cropped, sorted, and labeled to select 300 leaves per category and divided into a training and test set according to a 4:1 ratio. We amplified the images to 7500 using the imgAug library, which applied data augmentation by rotating the images by 90, 180, and 270 degrees and employing horizontal and vertical flips (**Table 2**). **Table 2** reports the details of the peanut disease leaves dataset. All images from this dataset are uniformly resized to 224×224 pixels before being input to the model.

**Table 2:** Basic information on peanut leaf disease.

Class	Train set	Test set
Healthy leaves (HL)	1200	300
Scorch disease (SD)	1200	300
Rust disease (RD)	1200	300
Both scorch and rust disease (SD+RD)	1200	300
Leaf-spot disease (LSD)	1200	300

### Operating environment and parameter setup

All trials are implemented on a Dell Precision 3640 PC (CPU I9-10900, 32GB RAM), utilizing an Nvidia GeForce RTX 2080 Super 8GB graphics card. Considering the software, we relied on Windows 10, Python 3.8.5, and Torch 1.9.0+cu102.

The subsequent trials utilize the VGG-16, ResNet-18, ResNet-50, and DenseNet-121 models, and the SENet, CBAM, ECA, and the improved ECA attention modules are added to ResNet-18. For a fair comparison of the models’ performance, we employ the same training parameters: the optimizer is the stochastic gradient descent (SGD), batch size of 32, 0.001 weight decay, 5e-4 learning rate, and the loss function is the CrossEntropyLoss. For the PlantVillage dataset, we consider 200 epochs, and for the peanut leaf disease dataset, 400 epochs.

### Workflow of the proposed method

#### Overview of the CACPNET approach

**Figure 2:**
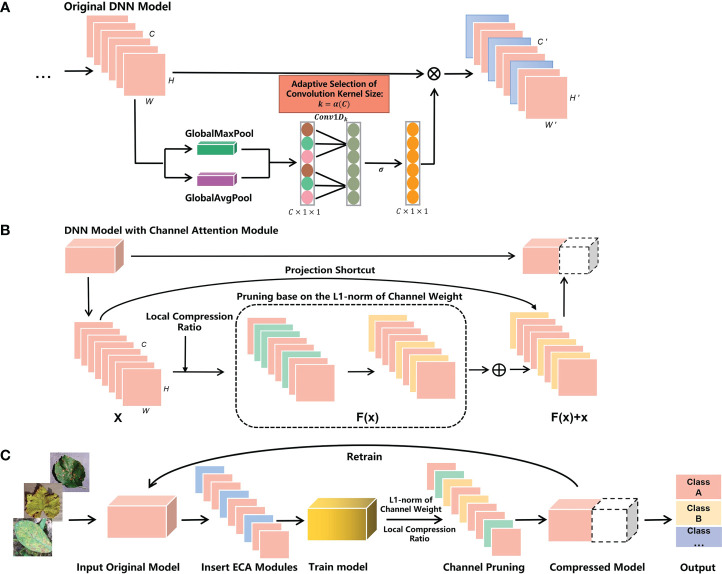
Workflow of the CACPNET. **(A)** Insert channel attention modules into the original model. The blue sections are the channel attention modules. Σ is the sigmoid activation function. **(B)** Use channel pruning to compress the DNN model. The green and yellow sections are the removed channels. **(C)** Overall structure of CACPNET.

**Figure 2A** illustrates the process of the channel attention module inserted into the model. Specifically, we traverse all model layers and insert the channel attention module after each convolutional layer. Then the new model is trained to achieve a better performance effect. Accordingly, **Figure 2B** depicts removing unimportant channels using channel pruning on the model. We obtain the weight relation in the channel from the well-trained model. Then the L1-normalization of the channel weights is calculated and ranked. The unimportant and associated channels per layer are removed based on a predetermined local compression ratio. Finally, the new model is updated with the remaining channels and retrained to achieve better performance.

**Figure 2C** illustrates the implementation process of CACPNET. In summary, this paper aims to develop a lightweight model with better performance and lower parameters, with the following sections introducing the details on implementing CACPNET.

### Improved ECA module

This paper’s channel attention module is based on an improved ECA module that uses a local cross-channel interaction strategy without dimensionality reduction. This strategy ensures that the information from the adjacent channels is correlated without losing image information, solving the correlation problem of the information contained in the plant leaf disease images. In summary, channel attention can be learned by:


(1) ω=σ(Wy)

which affords to gain the weight matrix (ω) if the output channels in the attention module. *W* is the parameter matrix (*C* × *C*) *y* is the channel attention operation, and σ is a sigmoid activation function of the channel attention module. The parameter σ is defined as follows:


(2) $$\sigma \left(x\right)=\frac{1}{1+e^{-x}}$$

After the DNN’s convolution operation, the output is the channel-independent parameter matrix (*C* × *C*) represented as:


(3) $$W=\left\{\begin{array}{c} W_{1}=\left[\begin{array}{ccc} w^{1,1} & \cdots & 0\\ \vdots & \ddots & \vdots \\ 0 & \ldots & w^{c,c} \end{array}\right]\\ \vdots \\ W_{i}=\left[\begin{array}{ccc} w^{1,1} & \cdots & w^{1,c}\\ \vdots & \ddots & \vdots \\ w^{c,1} & \ldots & w^{c,c} \end{array}\right] \end{array}\right\}$$

The convolution operations are performed on mutually independent channels, while the feature map information between the channels cannot interact. In order to achieve cross-channel interaction without dimensionality reduction, we use a band matrix *W_k_* f size *k* × *c*. Thus *W_k_* can be expressed as:


(4) $$W_{k}=\left[\begin{array}{cccccccc} w^{1,1} & \cdots & w^{1,k} & \cdots & 0 & \cdots & \cdots & w^{1,c}\\ 0 & w^{2,2} & \cdots & w^{2,k+1} & \vdots & \cdots & \cdots & 0\\ \vdots & \vdots & \vdots & \ddots & 0 & \vdots & \vdots & \vdots \\ w^{c,1} & 0 & 0 & \cdots & \cdots & w^{c,c-k+1} & \cdots & w^{c,c} \end{array}\right]$$

From expressions (3) and (4), we find that *W* contains one or more *W_k._*. However, *W_k_* avoids the problem of non-interacting channels between different groups in *W*. The range of cross-channel interactions depends on *k*. Nevertheless, the convolution of matrix *W_k_* requires frequent multiplication operations, increasing the model’s parameters and slowing down the running speed. Additionally, the attention mechanism operating on matrix *W_k_* is inefficient and will slow down the model. To preserve the model’s processing efficiency and to effectively obtain channel-wise feature information, we employ the ECA module, which uses a global averaging pooling (*G_avg_*) operation to retain the global information of each channel. However, global average pooling may discard the disease features since plant leaf diseases occur at small locations. Instead, the global max pooling (*G_max_*) can achieve translation invariance in the feature mapping and extract small location disease features (You et al., 2021). Therefore, we combine global average pooling and global max pooling affording the disease features to be effectively extracted while retaining the image’s global information. The global pooling expression is as follows:


(5) $$G\left(x\right)=G_{avg}\left(x\right)+G_{max}\left(x\right)=\frac{1}{H\times W}\sum_{i=1,j=1}^{HW}F\left(x\right)+\underset{1\leq i\leq H,1\leq j\leq W}{\max}F\left(x\right)$$

where *F*(*x*) is the aggregation feature of the input channels, and *HW* ( e the input channel shape (**Figure 2A**). After global pooling, the original multidimensional input *C* × *H × W* is transformed into a one-dimensional parameter matrix *C*×1×1 output. Therefore, a one-dimensional convolution kernel (*Conv*1*D*) can be used for efficient cross-channel information fusion operations, denoted as:


(6) $$\omega =\sigma \left(Conv1D_{k}\left(G\left(x\right)\right)\right)$$

where *k* is an adjustable parameter of the one-dimensional convolution kernel that determines the scope of the cross-channel interaction. Thus, there exists a mapping relationship *α* between the convolution kernel size *k* and channel *C* that can be expressed as:


(7) C=α(k)

### Channel pruning

This paper proposes a pruning method to remove unimportant channels from a well-trained model to reduce the model’s parameters and complexity. In the DNN model, the channels’ input and output between the layers are correlated. When the output channel of the upper layer is removed, the corresponding input channel of the lower layer also has to be removed. Therefore, the network structure of the model hierarchy must be appropriately built so that the input and output dimensions are consistent between the layers. The convolutional layer weight matrix of the DNN model consists of the input channels *C_in_* the output channels *C_out_* and the convolutional kernel size *H*×*W*. In this paper, the weight of the output channels is calculated and sorted to judge the channels’ importance, i.e., a pruning strategy for the L1-norm weights. It can be expressed as follows:


(8) $$C_{out}=\sum_{i=1}^{C_{in}}\sum_{i=1}^{H}\sum_{i=1}^{W}\sqrt{\omega^{2}}=\ell -1norm\left|\omega \right|$$

where ω is the weight matrix of the output channels, *ℓ*–1*norm*|*w*| σ is a squared summation of the weight matrix *w* to open the roots. i.e., converting the multidimensional weight matrix *C_in_* ×*H*×*W* into a one-dimensional weight matrix *C_out_*. The channels’ importance is obtained by sorting the weight matrix *C_out_*. Since each model layer has a different effect on the extracted image information, the shallow channels are more sensitive to model pruning than the deeper channels. Furthermore, over-pruning the shallow channels will seriously affect the model’s accuracy (Li et al., 2016). To reduce the influence of channel pruning on model accuracy, we introduce a local compression ratio *R* to prune different layers of the model. For different models and layers, the local compression ratio is an adjustable parameter, following the principle that the compression ratio of a shallow layer is smaller than a deeper layer. By introducing a local compression ratio, the model retains the crucial channels without significantly losing model accuracy and reduces the parameters and complexity. The removed channels are determined by applying L1-normalization on the weights and the local compression ratios. The number of removed channels is calculated as follows:


(9) $$P=R\times len\left(C_{out}\right),R\in \left[0,1\right),$$

where *P* is the number of channels removed in the current layer, *R* is the local compression ratio, and *len*(*C_out_*) is the number of the output channels. Although a larger compression ratio can reduce the model parameters and complexity, it significantly reduces the model’s accuracy. Considered together, the compression ratio of each layer for CACPNETD is set as *R*=[0, 0, 0.1, 0.1, 0.2, 0.2, 0.3, 0.3]. After removing *P* channels from this layer, the network structure in the DNN model is updated simultaneously, and the corresponding input channels of the next layer are removed.

It is worth noting that ResNet is a residual block with a shortcut connection. The expression for the residual block output *y* is:


(10) $$y=ℱ\left(x,\left\{W_{i}\right\}\right)+W_{s}x$$

In the ResNet model, the basic block, whose output comprises the convolutional layers’ output and the residual block’s output, contains two convolutional layers and a residual block. For the summation operation, the output dimension of the residual block must be the same as the convolutional layer. Therefore, the input *W_s_x* just fit the dimension of the residual learning function *F*(*x,{W_i_*}). He et al., 2016). However, several previous studies have not pruned for the residual block. To ensure the consistency between the residual block channels and convolutional layer output channels after pruning, we adopt a pruning parameter sharing strategy to solve the problem of pruning the residual block. The pruning equation of the residual block can be expressed as:


(11) $$P_{r}=O_{r}-O_{conv2},$$

where *P_r_* is the channel number removed by the residual block, *O_r_* is the output channel of the original residual block, and *O_conv_* is the output channel after pruning the previous convolutional layer. Finally, the weights after channel pruning are updated in the model.

### Combination of channel attention and channel pruning

We select ResNet-18 as the base model and traverse all its layers except the residual block layer. Moreover, we insert the improved ECA attention module after each convolutional layer. Training the model containing attention modules improves recognition accuracy and establishes the channel relationship. Additionally, the trained model employs the channel pruning operation mentioned above, and finally, the weights in the model are updated after channel pruning. The pruned model is retrained to achieve better performance, with the specific CACPNET implementation presented in **Algorithm 1**.

Algorithm 1. CACPNET Algorithm
**Input:**
*D* the dataset
*M* the original DNN model
*A* the ECA module
R the local compression ratios for each layer
**Output:**
ℳ the ECA model after pruning
**Procedure: for** each batch D_t_ ϵ*D*
**o end for for** each layer *l*ϵ*L*
**o end for**
Retraining model

### Evaluate metrics

We evaluate the model’s performance on the accuracy, F1 score, FLOPs, parameters, model size, and GPU RAM metrics. The accuracy and F1 score directly reflect the model’s recognition performance, while the F1 score is the summed average of precision and recall. In addition, the FLOPs, parameters, model size, and GPU RAM represent the model’s complexity and performance requirements of the running device. The model accuracy is expressed as:


(12) $$Accuracy=\frac{TP+TN}{TP+FP+TN+FN}$$

where *TP* is the prediction of positive classes as positive classes, *TN* is the prediction of negative classes as negative classes, *FP* is the prediction of negative classes as positive classes, and *FN* is the prediction of positive as negative classes.

The F1 score, precision, and recall are defined as:


(13) $$F1=\frac{2\times Precision\times Reacall}{Precision+Recall}$$

(14) $$Precision=\frac{TP}{TP+FP}$$

(15) $$Recall=\frac{TP}{TP+FN}$$

FLOPs stand for floating point operations, which are used to measure the model’s complexity, defined as:


(16) $$FLOPs=2\times k^{2}\times C_{in}\times h_{out}\times w_{out}\times C_{out}$$

where *k* is the convolution kernel size, *C_in_* the number of input channels, *h_out_* and *w_out_* are the height and width of the output channel, respectively, and *C_out_* the number of output channels.

The parameters of the model are related to the size of the convolution kernel and the number of feature Maps, calculated as follows:


(17) $$Parameters=C_{out}\times (C_{in}\times k^{2}+1)$$

## Results

### Ablation study on the improved attention module

**Figure 3:**
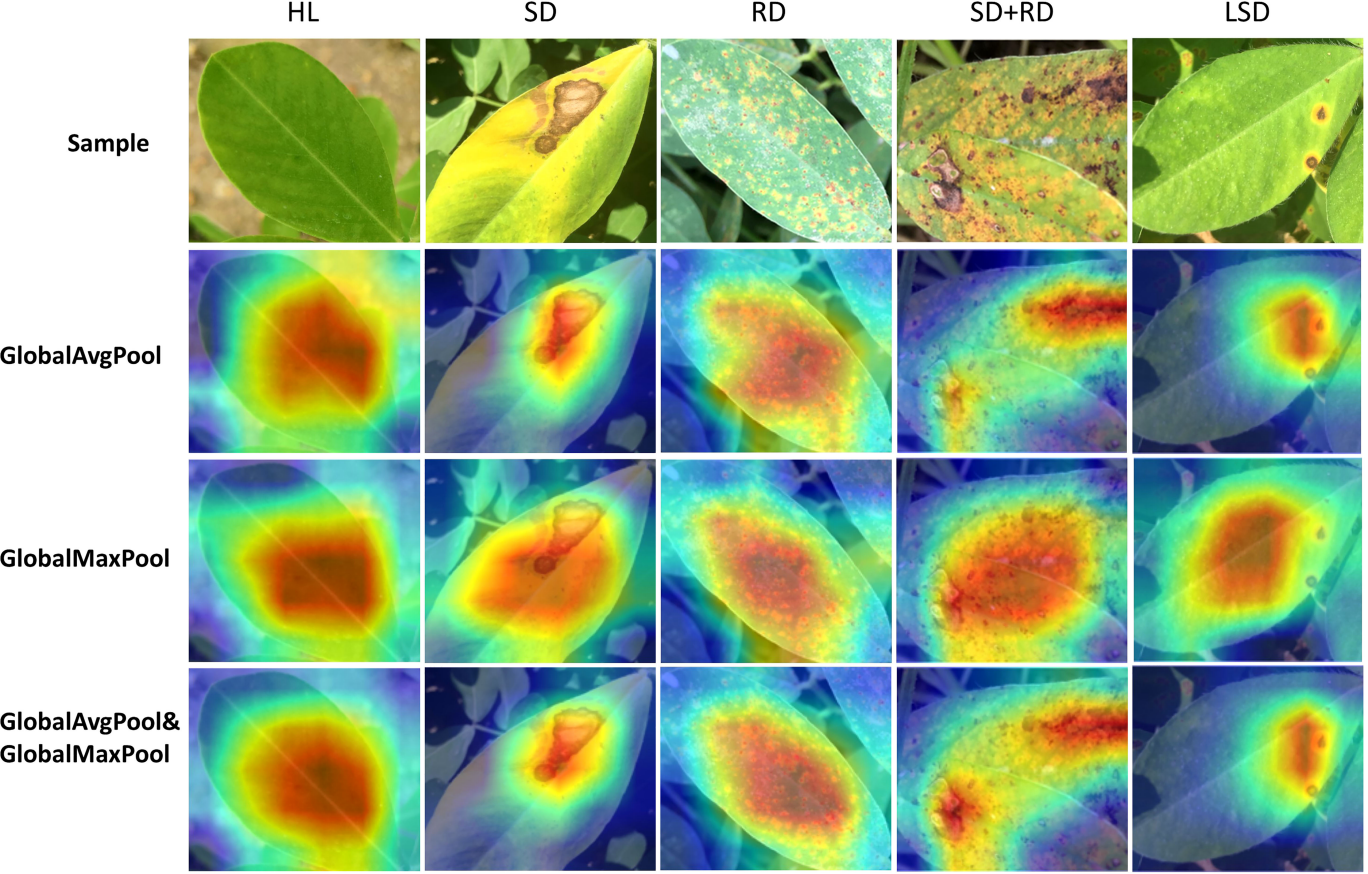
Visualized results of different pooling methods in recognizing peanut leaf disease. The dark color of the heat map represents the model’s focus.

The following experiments are on PlantVillage and peanut leaf disease datasets, while the model’s training parameters are described in detail in the materials and methods section. To compare the effects of global average pooling and global max pooling, the ECA module of different pooling methods is inserted into ResNet-18 (**Figure 2A**). The results highlight that using both global average pooling and global max pooling methods simultaneously achieves better accuracy (**Table 3**). The features learned by the model can be visualized using Grad-CAM (Selvaraju et al., 2020). The corresponding heat map reveals that using a pooling combination enables the model to focus on the disease features (**Figure 3**). Interestingly, the pooling method does not increase the model’s complexity, with the ablation results confirming that the proposed approach using the improved ECA module section is highly appealing. Therefore, global average and max pooling effectively extract the disease features (**Figure 3** and **Table 3**). Thus, the ECA module using global average pooling and global max pooling is used in the following experiments.

**Table 3:** Comparison of different pooling methods in channel attention.

Description	Accuracy of PlantVillage	Accuracy of peanut	GFLOPs	Parameter
ResNet-18	98.2%	95.1%	1.819	11.180M
ECA (GlobalAvgPool) & ResNet-18	99.6%	97.5%	1.819	11.180M
ECA (GlobalMaxPool) & ResNet-18	99.5%	97.4%	1.819	11.180M
ECA (GlobalAvgPool & GlobalMaxPool) & ResNet-18	99.7%	98.0%	1.819	11.180M

### Model training and validation on the PlantVillage

**Figure 4:**
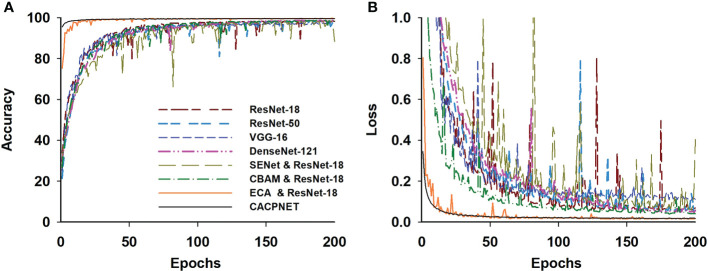
Model training and validation on the PlantVillage. **(A)** The accuracy curve of models in the PlantVillage test set; **(B)** The loss curve of models in the PlantVillage test set. Colors denote corresponding models.

This experiment challenges eight models, including the VGG-16, ResNet-18, ResNet-50, and DenseNet-121, and the remaining four are based on ResNet-18 assembled by adding SENet, CBAM, ECA, and the improved ECA attention module. All models are trained and validated using the PlantVillage dataset containing 14 species covering 38 classes of diseases (**Table 1**). The accuracy of all models increased along with iteration and converged at 200 epochs under the same training parameters (**Figure 4A**). The detailed data of each model are reported in **Table 4**. The accuracy of VGG-16 (97.5%) is the lowest among all models, and the parameter cardinality and FLOPs are the largest, demonstrating that a large parameter cardinality may increase the model’s computational effort without effectively improving accuracy. It is worth noting that the accuracy and F1 of ResNet-50 are higher than that of ResNet-18, indicating that increasing the depth of the model improves its accuracy. However, ResNet-50 has an increased value considering Flops, parameters, model size, and GPU RAM requirements (**Table 4**). In addition, DenseNet-121 attains an appealing accuracy but requires more GPU RAM (**Table 4**). Regarding the attention mechanism module, the ECA module has the fastest accuracy and loss curve convergence compared to SENet and CBAM (**Figure 4**). The model using the ECA module convergences well on the 50^th^ epoch. Moreover, the proposed model’s accuracy reaches 99.7%, which is the best among all competitor models. Compared with the original ECA model, CACPNET presents an increased accuracy based on the improved ECA attention module with channel pruning. Moreover, CACPNET has less accuracy and loss fluctuations during the training process, suggesting that it is more robust in various plants.

**Table 4:** Indicators of model performance.

Model	Accuracy of PlantVillage	F1 score of PlantVillage	Accuracy of peanut	F1 score of peanut	GFLOPs	Parameter	Model size	GPURAM
**ResNet-18**	98.2%	97.4%	95.1%	95.1%	1.819	11.180M	42.7M	2.4G
**ResNet-50**	98.6%	97.9%	95.3%	95.3%	4.109	23.518M	90.0M	4.4G
**VGG-16**	97.5%	96.8%	92.4%	92.4%	15.480	134.281M	512M	7.5G
**DenseNet-121**	98.7%	98.1%	95.9%	95.9%	2.865	6.959M	27.1M	6.0G
**SENet & ResNet-18**	98.5%	97.8%	95.6%	95.6%	1.820	11.266M	43.0M	2.4G
**CBAM & ResNet-18**	98.7%	98.2%	95.9%	95.9%	1.821	11.267M	43.1M	2.6G
**ECA & ResNet-18**	99.6%	99.4%	97.5%	97.5%	1.819	11.180M	42.7M	2.4G
**CACPNET**	99.7%	99.5%	97.7%	97.7%	1.267	4.699M	18.0M	2.2G

### Model training and validation on the peanut leaf disease dataset

**Figure 5:**
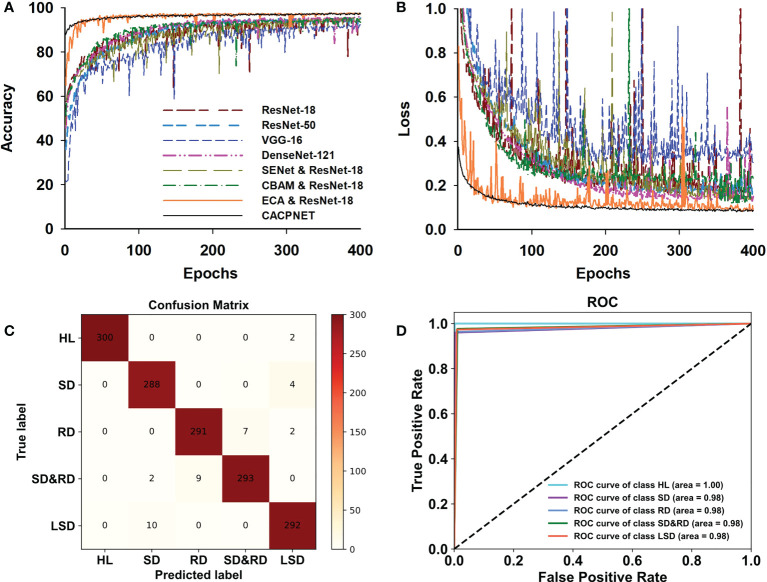
Model training and validation on the peanut leaf disease dataset. **(A)** The accuracy curve of models in the peanut test set. **(B)** the loss curve of models in the peanut test set. Colors denote corresponding models. **(C)** The confusion matrix of CACPNET in the peanut test set. The values in the corresponding column present the number of being predicted. The darker color indicates the larger quantity. **(D)** The ROC curve of CACPNET in the peanut test set. Each curve represents the identification effect of the corresponding class in the model. The closer to the upper left corner of the curve means better recognition for the corresponding class.

When applying CACPNET on the PlantVillge dataset, it obtains better accuracy and F1 score after channel pruning and using the ECA module on 14 species (**Figure 4** and **Table 4**). To verify the robustness of CACPNET further, the subsequent trials utilize the peanut dataset collected from an actual environment. A detailed description of the dataset acquisition is presented in the Materials and Methods section. All models converge after 400 training epochs (**Figures 5A, B**). However, the accuracy and F1 score of all models on the peanut test set is slightly lower than the PlantVillage (**Table 4**), potentially due to image interference factors originating from the actual environment. Nevertheless, the CACPNET’s identification accuracy still reaches 97.7%, outperforming all competitor models. Furthermore, the accuracy and loss curves of the competitor models fluctuate more than CACPNET (**Figures 4**, **5A, B** and **Table 4**), reconfirming our method’s robustness.

However, rust and scorch diseases may often appear on the same leaf, easily leading to misidentification or incomplete identification. Thus, accurately identifying the two diseases when in a mixture is difficult in actual agricultural situations. However, CACPNET maintains a good identification performance, with the confusion matrix indicating a high prediction accuracy and a low error (**Figure 5C**). Regarding image classification identification, the ROC curves per identification class are considered a binary classification problem. The closer the curve to the upper left corner, the better the learning performance (**Figure 5D**). CACPNET achieves an area under the ROC curve of at least 0.98 for all five peanut leaf disease identifications (**Figure 5D**). In summary, CACPNET is more robust and performs better for specific class identification than the competitor methods.

### Analysis of the model performance index

**Figure 6:**
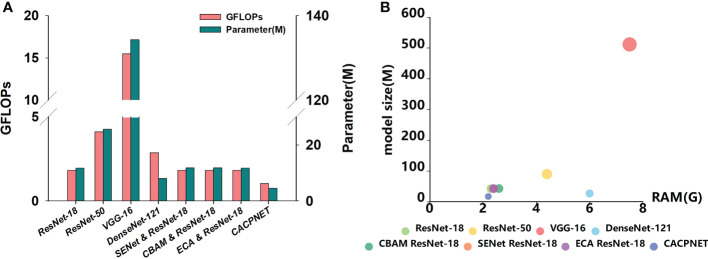
Analysis of model performance index. **(A)** Histogram of FLOPs and parameters for eight models; **(B)** Bubble chart of model size and GPU RAM for eight models.

The previous results indicated that CACPNET performed great in both datasets considering accuracy and F1 score (**Table 4**). Additionally, the FLOPs, parameters, model size, and GPU RAM are essential metrics to evaluate the model’s performance. FLOPs stand for floating point operations and are used to measure the complexity of a model, while the model’s parameters directly determine the model’s size and memory requirements. Additionally, the model’s size and GPU RAM are the model’s direct reflection of the required physical memory. By comparing the FLOPs and parameters among all models, we find that the FLOPs of ResNet-18 are smaller than ResNet-50, VGG-19, and DenseNet-121, while the parameters of ResNet-18 are higher than DenseNet-121 (**Table 4** and **Figure 6A**). When adding the SENet and CBAM attention modules to ResNet-18, the FLOPs and parameter cardinality slightly increase, while FLOPs and parameters are the same when adding the ECA module (**Table 4** and **Figure 6A**).

Although the ECA module effectively improves the model’s identification accuracy without increasing its parameters and complexity (**Figures 4**, **5**, **6A** and **Table 4**), the parameters and complexity still limit the model from being deployed in edge devices with limited computational capabilities. Therefore, to decrease the parameters and complexity, we apply channel pruning to the model based on the improved ECA module. The FLOPs and parameters of CACPNET are significantly lower than ResNet-18 after channel pruning (**Table 4** and **Figure 6A**). Interestingly, we maintain a high identification accuracy despite reducing the model’s parameters and FLOPs through channel pruning. The model size and GPU RAM requirements of all models are presented in **Figure 6B**. Among all models, CACPNET has the minimum requirements on both model size and GPU RAM. Compared to ResNet-18, the FLOPs, parameters, model size, and GPU RAM of CACPNET decreased by 30.35%, 57.97%, 57.85%, and 8.3%, respectively (**Table 4**). In brief, without any reduction in accuracy and the F1 score, CACPNET achieves a significant reduction in FLOPs, parameters, model size, and GPU RAM. Therefore, CACPNET is more appealing according to the performance metrics.

### Identification rate evaluation of the model

**Figure 7:**
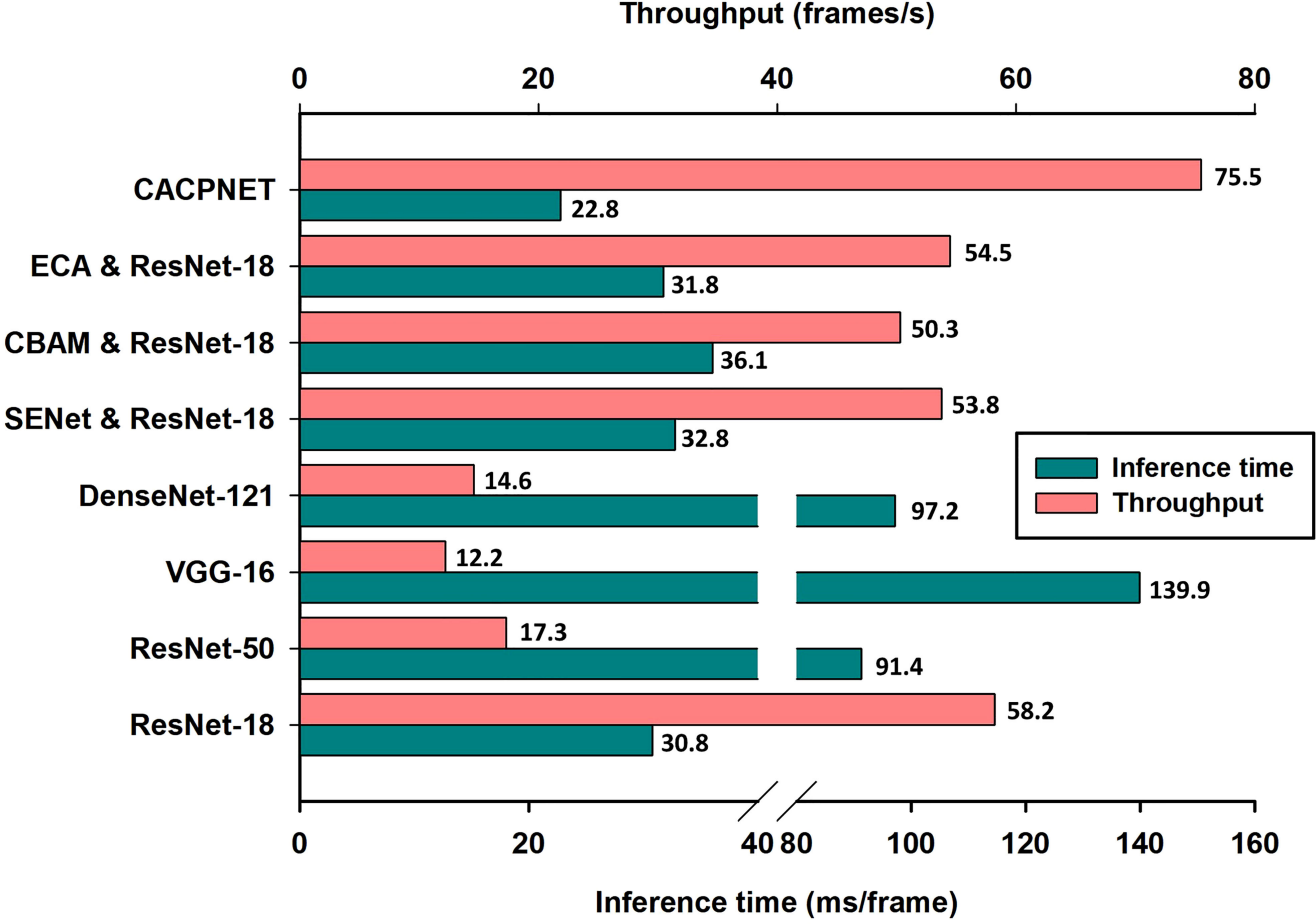
The inference time and throughput of the model. An i9-10900 was used as the CPU throughout the experiments.

In the plant disease detection scenarios, the computational performance of the plant protection devices is limited, generally relying on the CPU for the computations. To validate the model’s identification rate, we deployed all models for disease identification on the CPU. The metrics employed are the inference time and throughput, two important performance recognition metrics representing the time required to recognize each image and the number of images that can be processed per unit time, respectively. The experimental results highlight that the inference time and throughput of CACPNET outperform the competitor models, i.e., 22.8 ms/frame and 75.5 frames/s, respectively (**Figure 7**). Compared with the baseline model ResNet-18, adding the ECA, SENet, and CBAM attention modules reduces the model’s recognition rate (**Figure 7**). However, the proposed CACPNET improves recognition accuracy and increases recognition rate (**Figure 7** and **Table 4**). The above experimental data prove that CACPNET can operate efficiently in plant protection equipment.

## Discussion

This study proposes a plant leaf disease identification model (CACPNET) that combines a channel attention mechanism and channel pruning. The PlantVillage and peanut leaf disease dataset results reveal that CACPNET’s recognition accuracy and F1 score are the highest among all models (**Table 4**; **Figures 4**, **5A, B**). Moreover, CACPNET has the most appealing (lowest) performance factors, such as FLOPs, parameters, model size, and GPU RAM (**Table 4**; **Figure 6**), proving that CACPNET is a lightweight model with high recognition accuracy.

Although using attention mechanisms in deep learning has made significant progress in image identification (Woo et al., 2018; Hu et al., 2019; Wang et al., 2020), its modules have not been designed for plant diseases in specific. Identification errors cannot satisfy the disease control requirements of precision agriculture. Therefore, this paper improves the ECA attention module to combine global maximum pooling with global average pooling, effectively improving the model’s disease feature extraction ability.

The attention mechanism can improve the mode’s recognition accuracy. However, models containing many parameters are unsuitable for planting equipment deployment with limited computing power. In particular, using the attention mechanism reduces the model’s recognition and fails to meet the requirements of real-time detection regarding inference time and throughput metrics. Although the model can be simplified by channel pruning, the identification accuracy will significantly decrease due to losing important parameters (Guo et al., 2020).

Based on the existing problems presented above, we remove the unimportant channels by introducing a local compression ratio and an L1-norm channel weight to reduce the model’s complexity and parameters. However, compared with other models, the channel-pruned CACPNET still attains the highest accuracy (**Table 4**; **Figures 3**–**5**). It is worth noting that the local compression ratio is a critical parameter that impacts the model’s pruning effect. Although an excessive local compression ratio can significantly reduce the model’s parameters and complexity, it also reduces accuracy. Therefore, it is necessary to set a reasonable local compression ratio for model pruning that depends on the model. The proposed method can maintain the model with high accuracy and lightweight at the same time, which has excellent generality to be applied to other models. In this paper, the purpose of using ResNet-18 as the base model is to obtain a more lightweight and higher accuracy model on a shallow network.

Currently, studies exist on wheat (Mi et al., 2020), apples (Yong and Ming, 2020), and grapes (Xie et al., 2020) but are constrained to a particular plant lacking universality. Unlike current methods, CACPNET is challenged on the PlantVillage database that contains 14 crops and 38 diseases, affording an identification accuracy after training of 99.7%, indicating that CACPNET can accurately identify different species and the disease characteristics of each species (**Tables 1**, **3**, **4**; **Figure 4**). To further verify the disease feature extraction ability of CACPNET, the leaves of peanut diseases collected in actual fields are used for training, including leaves with complex diseases such as leaves with both scorch and rust (**Figure 1**). Surprisingly, the identification accuracy of CACPNET reaches 97.7%, demonstrating its excellent disease feature extraction (**Tables 3**, **4**; **Figure 5**).

In the precision agriculture field, real-time disease detection is an effective way to detect and control diseases in a timely manner. Inference time and throughput are important metrics to measure the real-time recognition rate of the model. Since CACPNET has a significant lead in FLOPs and parameter metrics (**Figure 6** and **Table 4**), CACPNET is also ahead of other models in terms of inference time and throughput on CPU-based devices, with 22.8 ms/frame and 75.5 frames/s, respectively (**Figure 7**). The above results demonstrate that the recognition rate of CACPNET can satisfy the requirement of real-time disease detection.

Some advances in leaf disease identification have been made utilizing hyperspectral imaging (Ban et al., 2019; Nagasubramanian et al., 2019). However, the high weather or light requirements, professional operation, and extra-expensive equipment limit its overall development. Opposing, the proposed model has low image resolution requirements (224×224 or higher resolution) derived from mobile phones and pads. 

## Conclusion

This study proposes a lightweight model named CACPNET that is based on channel attention and channel pruning. Compared with other models, CACPNET has prominent advantages. First, CACPNET has the highest accuracy and F1 score among all competitor methods. Second, CACPNET’s ability to extract plant leaf disease features can be effectively improved by combining global average pooling and global maximum pooling. In addition, CACPNET outperforms other models considering the parameters, FLOPs, Model size, and GPU RAM performance metrics. For devices relying on the CPU as the computing core, the inference time and throughput of CACPNET are superior to other models and still meet the real-time identification requirement. To sum up, CACPNET is a lightweight and highly accurate model for plant leaf disease recognition that is appropriate for lightweight model deployment in the plant protection field and promotes the development of artificial intelligence in precision agriculture. Meanwhile, this study fills the research gap in real-time detection of leaf diseases, including peanuts, potatoes, apples, and other 15 crops and 43 diseases, providing the basis for decision-making in precision agriculture.

In future work, we plan to deploy CACPNET to field robots and unmanned aerial vehicle to establish an automated disease detection platform with low inference cost. In addition, to extend CACPNET’s applicability on disease identification of other plants, we will consider expanding its disease identification types through transfer learning.

## Data availability statement

Publicly available datasets were analyzed in this study. This data can be found here: https://plantvillage.psu.edu/.

## Author contributions

RC: Conceptualization, Methodology, Software, Data curation, Visualization Writing- original draft, review, editing and revision. HQ: conceptualization, methodology, resources, review and editing, project administration and supervision, funding acquisition. YL: data curation, validation, formal analysis, investigation. MY: visualization, editing, revision. All authors contributed to the article and approved the submitted version.

## Funding

This research is funded by Characteristic innovation projects of Guangdong Provincial Department of education in 2019 (2019KTSCX015), Key-Area Research and Development Program of Guangdong Province (2019B020214005), The National Key Research and Development China Subproject (2021YFD2000701).

## Acknowledgments

The authors would like to express their gratitude to EditSprings (https://www.editsprings.cn) for the expert linguistic services provided.

## Conflict of interest

The authors declare that the research was conducted in the absence of any commercial or financial relationships that could be construed as a potential conflict of interest.

## Publisher’s note

All claims expressed in this article are solely those of the authors and do not necessarily represent those of their affiliated organizations, or those of the publisher, the editors and the reviewers. Any product that may be evaluated in this article, or claim that may be made by its manufacturer, is not guaranteed or endorsed by the publisher.
